# Schizophrenia: susceptibility genes and oligodendroglial and myelin related abnormalities

**DOI:** 10.3389/fncel.2014.00005

**Published:** 2014-01-21

**Authors:** Panos Roussos, Vahram Haroutunian

**Affiliations:** ^1^Mental Illness Research, Education, and Clinical Center (VISN 3), James J. Peters VA Medical CenterBronx, NY, USA; ^2^Department of Psychiatry, Icahn School of Medicine at Mount SinaiNew York, NY, USA; ^3^Department of Genetics and Genomic Science and Institute for Multiscale Biology, Icahn School of Medicine at Mount SinaiNew York, NY, USA; ^4^Department of Neuroscience, Icahn School of Medicine at Mount SinaiNew York, NY, USA

**Keywords:** systems biology, polygenic, node of Ranvier, disconnectivity, GWAS

## Abstract

Given that the genetic risk for schizophrenia is highly polygenic and the effect sizes, even for rare or *de novo* events, are modest at best, it has been suggested that multiple biological pathways are likely to be involved in the etiopathogenesis of the disease. Most efforts in understanding the cellular basis of schizophrenia have followed a “neuron-centric” approach, focusing on alterations in neurotransmitter systems and synapse cytoarchitecture. However, multiple lines of evidence coming from genetics and systems biology approaches suggest that apart from neurons, oligodendrocytes and potentially other glia are affected from schizophrenia risk loci. Neurobiological abnormalities linked with genetic association signal could identify abnormalities that are more likely to be primary, versus environmentally induced changes or downstream events. Here, we summarize genetic data that support the involvement of oligodendrocytes in schizophrenia, providing additional evidence for a causal role with the disease. Given the undeniable evidence of both neuronal and glial abnormalities in schizophrenia, we propose a neuro-glial model that invokes abnormalities at the node of Ranvier as a functional unit in the etiopathogenesis of the disease.

## INTRODUCTION

The core symptoms of schizophrenia include the presence of delusions and hallucinations (positive symptoms), apathy, and social withdrawal (negative symptoms), as well as, stable impairments in specific domains of cognitive function. Despite our best effort, we still lack a basic understanding of the etiopathogenesis of schizophrenia and therefore tools for curative treatment or prevention do not exist (Insel, 2010). Given the high prevalence of schizophrenia (1% or more) the need for greater understanding is urgent.

Most efforts in understanding the cellular basis of schizophrenia have followed a “neuron-centric” approach, focusing exclusively on the role(s) of neurons. This has led to findings of alterations in neuronal function, such as neurotransmission and synapse cytoarchitecture, often overlooking the fact that neuronal function and neurotransmission is inextricably dependent on interactions between neurons and glia. Increasing recognition of the importance of neuro-glial elements as basic functional units (Allen and Barres, 2009) that participate in CNS function and dysfunction combined with multiple lines of evidence showing abnormalities in oligodendrocytes and myelination have implicated oligodendrocytes as highly relevant to schizophrenia. While the approach of grouping neurons and glia into neuro-glial functional units introduces significant complexity into the analysis of the neurobiology of schizophrenia, it encourages an integrative approach that favors the examination of the interplay between neurons and the more abundant glial cell types of the CNS.

Here, we first outline genetic evidence derived from candidate gene association studies or genome-wide approaches that support oligodendroglial and myelin related (OMR) abnormalities in schizophrenia as a primary contributor. In an attempt to provide a mechanistic and integrative interpretation of the neuronal and OMR changes in schizophrenia, we introduce a neuro-oligodendroglial model that involves abnormalities at the nodes of Ranvier (NOR) as a functional unit implicated in the etiopathogenesis of the disease.

## MYELIN ASSOCIATED ABNORMALITIES IN SCHIZOPHRENIA

Myelination can increase action potential transmission speed and decrease refractory time, which increases the number of action potentials that can be transmitted per unit time. There is accumulating evidence supporting the notion of schizophrenia as a disorder of disconnectivity (Konrad and Winterer, 2008). While disconnectivity can be caused by different mechanisms, such as synaptic malfunction, at its core, it implicates inadequate or failed information transfer among neurons. OMR abnormalities in schizophrenia impair the saltatory conduction and information conduction from one neuron to others.

Growing evidence, accumulated over the last 15 years, provide strong support for OMR abnormalities in schizophrenia. The majority of those data is derived from neuroimaging and human postmortem studies (for review, see Sequeira et al., 2012; Fitzsimmons et al., 2013). Irrespective of the level of care and control, different confounding factors, such as antipsychotic medication, could contribute to the detected OMR neuroimaging, gene expression, and proteome abnormalities in schizophrenia. Therefore, incorporating genetic data, as discussed below, can help distinguish whether OMR changes are a primary contributor to disease pathophysiology vs. secondary changes associated with other primary lesions existing in schizophrenia or mere epiphenomena.

### GENETIC ASSOCIATION OF OMR CANDIDATE GENES IN SCHIZOPHRENIA

Multiple candidate studies have provided evidence that OMR genes are genetically associated with schizophrenia (**Table 1**). It is interesting to note, that the products of these same genes have been implicated in numerous direct studies of OMR gene and protein expression in the brains of persons with schizophrenia, as well as, neuroimaging studies, providing a stronger support for causality with the disease (Bartzokis, 2011; Takahashi et al., 2011). The OMR genes with the strongest support for genetic association with schizophrenia are described below:

**Table 1:** OMR genes implicated in schizophrenia.

Gene	Genetic evidence	PGC-SWE P	Rank	Altered gene expression	Altered proteome
*ANK3*	Athanasiu et al. (2010), Roussos et al. (2012a), Yuan et al. (2012b)	1.31E-05	2	Roussos et al. (2012a)	Cruz et al. (2009), Roussos et al. (2012a)
*CLDN11*		9.04E-05	5	Tkachev et al. (2003), Katsel et al. (2005), Dracheva et al. (2006), Roussos et al. (2012a,b)	
*CNP*	Peirce et al. (2006), Voineskos et al. (2008)	3.94E-03	19	Hakak et al. (2001), Katsel et al. (2005), Dracheva et al. (2006), McCullumsmith et al. (2007), Barley et al. (2009), Roussos et al. (2012b)	Prabakaran et al. (2004), Dracheva et al. (2006)
*CNTNAP2*	Friedman et al. (2008), Wang et al. (2010), Ji et al. (2013)	3.95E-04	10	Roussos et al. (2012a)	
*DISC1*	Millar et al. (2000), Saetre et al. (2008), Song et al. (2008), Rastogi et al. (2009), Ayalew et al. (2012), Debono et al. (2012)	1.04E-03	13	Nakata et al. (2009)	Ratta-Apha et al. (2013)
*ERBB3*	Li et al. (2009)	9.70E-05	6	Tkachev et al. (2003), Katsel et al. (2005), Roussos et al. (2012b)	
*ERBB4*	Norton et al. (2006), Silberberg et al. (2006), Agim et al. (2013)	2.80E-06	1	Silberberg et al. (2006), Law et al. (2007)	Chong et al. (2008)
*MAG*	Wan et al. (2005), Yang et al. (2005)	1.96E-03	16	Tkachev et al. (2003), Katsel et al. (2005), Dracheva et al. (2006), McCullumsmith et al. (2007), Barley et al. (2009), Roussos et al. (2012b)	
*MAL*		8.23E-03	21	Hakak et al. (2001), Katsel et al. (2005)	
*MBP*	Ayalew et al. (2012)	4.23E-04	11	Tkachev et al. (2003), Matthews et al. (2012)	Martins-de-Souza et al. (2009a,b)
*MOBP*	Ayalew et al. (2012)	6.65E-03	20	Tkachev et al. (2003)	
*MOG*	Liu et al. (2005)	1.00E-02	23	Tkachev et al. (2003), Katsel et al. (2005), Barley et al. (2009), Roussos et al. (2012b)	Martins-de-Souza et al. (2009a)
*NFASC*		2.57E-04	8	Roussos et al. (2012a)	
*NRCAM*	Ayalew et al. (2012)	1.14E-03	14	Roussos et al. (2012a)	
*NRG1*	Stefansson et al. (2002), Ayalew et al. (2012), Weickert et al. (2012), Agim et al. (2013)	3.24E-05	3	Hashimoto et al. (2004), Law et al. (2006), Tan et al. (2007)	Chong et al. (2008)
*OLIG2*	Georgieva et al. (2006)	2.13E-03	17	Tkachev et al. (2003), Katsel et al. (2005)	
*PLP1*	Qin et al. (2005)	NA	NA	Tkachev et al. (2003)	
*PMP22*	Kirov et al. (2009)	1.38E-03	15	Katsel et al. (2005), Dracheva et al. (2006)	
*PTPRZ1*	Buxbaum et al. (2008)	6.21E-05	4		
*QKI*	Aberg et al. (2006b), Ayalew et al. (2012)	1.08E-04	7	Katsel et al. (2005), Aberg et al. (2006a), McCullumsmith et al. (2007)	
*RTN4R*	Budel et al. (2008)	6.75E-04	12		
*SOX10*	Maeno et al. (2007), Yuan et al. (2012a)	8.24E-03	22	Tkachev et al. (2003), Katsel et al. (2005), Dracheva et al. (2006)	
*CNTN2*		2.57E-04	8	Roussos et al. (2012a),b	
*TF*	Qu et al. (2008)	2.25E-03	18	Hakak et al. (2001), Katsel et al. (2005), McCullumsmith et al. (2007), Roussos et al. (2012b)	Prabakaran et al. (2004)

*The largest PGC-SWE (Ripke et al., 2013) in the form of summary statistic *p*-values was obtained from public access website (https://pgc.unc.edu/). For each gene (±100 kb from 5′ and 3′ ends of gene), the strongest *P* value association is provided in the “PGC-SWE *P*” column. The “Rank” column indicates the ranking of the illustrated genes based on the strength of association with the schizophrenia GWAS data set.*

#### Neuregulin 1 (NRG1) – ERBB4 signaling

*NRG1* risk genotypes or haplotypes have been associated with schizophrenia (Stefansson et al., 2002). Genetic evidence also supports *ERBB4* – the *NRG1* receptor – as a candidate susceptibility gene and suggests positive epistatic interactions between *NRG1* and *ERBB4* in schizophrenia (Norton et al., 2006). The potential pathophysiologic role of *NRG1* is further supported by its diverse neurobiological functions, including neuro-glial trophic effects and myelination (Harrison and Law, 2006). Nevertheless, given the fact that the *NRG1* – *ERBB4* signaling has multiple effects in nervous system development (including neuronal migration and modulation of neurotransmission), this pathway may play a role in schizophrenia pathogenesis through other mechanisms aside from OMR effects. A direct way to assess the pathophysiologic effects of genetic variants would be to examine human subjects with risk variants for symptoms of schizophrenia. Along these lines, many groups have reported association of the *NRG1* – *ERBB4* risk variants with neurocognitive (Stefanis et al., 2007), electrophysiological (Roussos et al., 2011) and neuroimaging schizophrenia-related outcome variables, including altered fronto-temporal brain function (Hall et al., 2006) and white matter density and integrity (McIntosh et al., 2008; Konrad et al., 2009). Finally, several groups have assessed gene expression and proteins of *NRG1* and *ERBB4* in postmortem brains and have reported differences between schizophrenia and healthy control groups (Hashimoto et al., 2004; Law et al., 2006).

#### Disrupted-in-schizophrenia 1

*Disrupted-in-schizophrenia 1 (DISC1)* is a strong candidate gene for schizophrenia and was initially identified in a large Scottish pedigree (Millar et al., 2000) followed by additional genetic evidence for association with sporadic cases of schizophrenia (Chubb et al., 2008). Recent studies in zebrafish (Drerup et al., 2009) and transgenic mice (Katsel et al., 2011) with forebrain restricted expression of mutant human *DISC1* have suggested a critical role for *DISC1* in oligodendroglial differentiation during neurodevelopment. In human postmortem analyses, *DISC1* transcripts that are more abundant during fetal development are upregulated in the hippocampus of patients with schizophrenia and their expression levels are associated with schizophrenia risk *DISC1* polymorphisms (Nakata et al., 2009). One of those risk *DISC1* SNPs is also associated with white matter integrity as measured by DTI (Sprooten et al., 2011).

#### Reticulon 4 receptor

*Reticulon 4 receptor (RTN4R)* is a myelin-associated protein that inhibits the outgrowth of neurites and nerve terminals (Chen et al., 2000). *RTN4R* is upregulated in the brains of patients with schizophrenia (Novak et al., 2002). Genetic evidence supports a role of *RTN4R* in the etiopathogenesis of schizophrenia (Novak et al., 2002; Budel et al., 2008). Interestingly, *in vitro* experiments demonstrated that cultured neurons expressing the schizophrenia-associated *RTN4R* variants failed to respond to the growth inhibiting activity of myelin, and functioned as a dominant negative to disrupt endogenous *RTN4R *(Budel et al., 2008).

#### Other genes

Oligodendrocyte lineage transcription factor 2 (*OLIG2*) encodes a transcription factor central to oligodendrocyte development (Ross et al., 2003). Genetic association analysis showed that *OLIG2* is associated with schizophrenia and demonstrated an epistatic effect with 2′,3′-cyclic nucleotide 3′-phosphodiesterase (*CNP*) and *ERBB4 *(Georgieva et al., 2006). Furthermore, *OLIG2* expression significantly correlated in cerebral cortex with *CNP* and *ERBB4*, suggesting that variation in *OLIG2* confers susceptibility to schizophrenia as part of a network of genes implicated in oligodendrocyte function. Similarly, a genetic association of the CNP gene was found for schizophrenia (Peirce et al., 2006). The CNP risk polymorphism was associated with lower gene expression which is consistent with CNP gene expression downregulation in schizophrenia.

### GENETIC ASSOCIATION OF OMR GENES IN SCHIZOPHRENIA DERIVED FROM GENOME-WIDE APPROACHES

Significant progress has been made over the last decade through large genome-wide association studies (GWAS). The GWAS design has provided evidence in an agnostic manner that specific common DNA genetic variants among people influence their genetic susceptibility to multiple different complex disorders, including schizophrenia (Ripke et al., 2013). Furthermore, we have learned that the genetic risk for schizophrenia is highly polygenic (Ripke et al., 2013), where the phenotype is influenced by multiple genetic variants, each one with small effect sizes. Here, we present results for association of each OMR gene with schizophrenia according to the largest published schizophrenia GWAS data set (PGC-SWE;https://pgc.unc.edu/; Ripke et al., 2013). No OMR genetic association reached genome-wide significance (**Table 1**); however, a handful of OMR genes (*ANK3, ERBB4, *and* NRG1*) show suggestive association (*P* < 5 × 10^-5^). Future GWAS with increased sample sizes will determine whether OMR genetic variants are true associations at reduced thresholds, but without the needed power to reach genome-wide significance.

**FIGURE 1:**
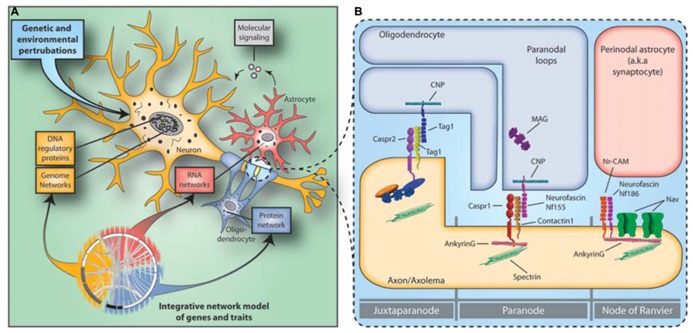
**Neuro-glial functional unit and the disconnectivity syndrome in schizophrenia.** **(A)** Constellations of multiple genetic and environmental perturbations affect molecular states of gene coexpression and protein–protein interaction networks in the neuro-glial functional unit that in turn increase the risk for schizophrenia. **(B)** The NOR is the site of intimate neuroglial cell–cell interactions in the nervous system. Abnormalities in the expression of NOR genes and proteins in schizophrenia hamper saltatory conduction by affecting NOR formation, maintenance and integrity and lead to the disconnectivity syndrome.

Systems biology approaches allow the integration of multi-scale datasets gained from genetic imaging and gene and protein expression studies. The risk SNPs are unlikely to be randomly distributed, but cluster in molecular subnetworks affecting specific cellular populations and molecular processes (Roussos, 2012; **Figure 1A**). Recent findings support the notion that polygenic risk for schizophrenia affects OMR gene networks and molecular pathways (Roussos et al., 2012b; Goudriaan et al., 2013). Perturbations in specific subnetworks related to neuronal function, myelination, immune response, and energy production were identified in a multi-regional system-level analysis of the schizophrenia brain transcriptome (Roussos et al., 2012b). The subnetworks related to neuronal function and myelination were enriched for genetically associated variants in schizophrenia, providing independent support for the neuro-oligodendroglial interactions as a potential causal functional unit in schizophrenia. No enrichment for genetic variants and immune response and energy production related subnetworks was found, suggesting that these changes may represent secondary or bystander phenomena or be attributable to environmental influences. Similar results were reported in an independent study, in which the genetic evidence for primary roles of specific glial cell type functions and pathways was examined based on gene set analysis (Goudriaan et al., 2013). In conclusion, recent systems biology approaches support the notion that schizophrenia involves hundreds of genetic loci that in combination with environmental factors converge to perturb a more selective/restrictive set of molecular/cellular networks and integrative functions.

## FUNCTIONAL NEURO-GLIAL UNITS AND THE NODES OF RANVIER

The above observations suggest that genetic alterations underlying OMR cell type functions increase susceptibility to schizophrenia and provide evidence that the “neuron-centric” hypothesis of schizophrenia should be extended to include a role for glia in the etiopathogenesis of the disease. The NOR is one of the best example of the neuroglial cell–cell interactions in the central nervous system. The NORs are myelin-sheath-free segments along myelinated axons, where sodium ion exchange takes place propagating action potentials from the axon initial segment to the axon terminal. NOR and OMR gene and protein expression abnormalities can hamper saltatory conduction by affecting NOR formation, maintenance, and integrity, which in turn leads to failures of saltatory conduction and disconnection of higher-order association areas (Nave, 2010).

Different and specific neuronal and oligodendroglial reciprocal interactions (for review, see Poliak and Peles, 2003) ensure the high concentration and anchoring of voltage gated Na^+^ channels (Na_V_; Na_V_1.6 in adults – *SCN8A*) to the NOR and the maintenance of adherens junctions between the axolemma and paranodal myelin loops (**Figure 1B**). The voltage gated Na^+^ channel cytoplasmic loops are linked to the underlying spectrin-actin cytoskeleton through the anchoring protein Ankyrin G (*ANK3*). *ANK3* plays an important role in the NOR complex by anchoring not only the Na_V_ to the node but also because several *cis *acting nodal and paranodal adherens junction proteins, such as neurofacin (*NFASC*), neuronal cell adhesion molecule (*NRCAM)*, contactin 1 and caspr, are also associated with it. Finally, contactin 2 (*CNTN2*), along with Caspr interact *in trans* with cell adhesion molecules of oligodendroglial paranodal loops that include an isoform of *NFASC* (Nf155), and *CNTN2* to ensure the integrity of the myelin-axolemma tight junctions, which form a barrier that prevents the diffusion of Nav from the nodal region.

A recently study demonstrated that several NOR genes and proteins, including *ANK3*, *NFASC, NRCAM, CNTN2*, and *SCN8A* are affected in schizophrenia (Roussos et al., 2012a). The importance of *ANK3* to serious mental illness is further supported by recent GWAS implicating *ANK3* in schizophrenia (Athanasiu et al., 2010) and bipolar disorder (Sklar et al., 2011). Furthermore, an *ANK3* risk polymorphism is associated with decreased *ANK3* gene expression, which is consistent with *ANK3* gene expression downregulation in schizophrenia and neurocognitive and neuroimaging abnormalities (Roussos et al., 2012a). Overall, these data highlight that in addition to OMR changes in schizophrenia, abnormalities in how myelin sheaths interact with the axon to form the NOR are also present and identify the NOR as a functional neuro-glial unit where neuronal and OMR genetic perturbations could potentially converge.

## Conclusion

There are multiple lines of evidence derived from neuroimaging, molecular and genetic data that support a role of OMR genes and molecular pathways in schizophrenia. Recent systems biology and pathway analyses that integrate gene expression and genetic data support the notion that oligodendroglial abnormalities are among the primary deficits in the disorder. Future studies need to integrate the larger GWAS and determine the polygenic effect of OMR genes in schizophrenia. While most efforts in understanding the cellular basis of schizophrenia have followed a “neuron-centric” approach, there is an emerging need to conduct more complex neurobiological studies and examine the neuro-glial unit as a whole. The data to date provide evidence for abnormalities in the NOR as a plausible biological substrate for the disconnectivity syndrome in schizophrenia. Recent evidence suggesting that antipsychotics may influence OMR function beneficially provides clues for improved treatment strategies (Ren et al., 2013). Therefore, a more thorough understanding of the role of OMR and NOR pathophysiology is necessary since it holds the potential for providing new insights into the treatment of this disease.

## Author Contributions

Panos Roussos and Vahram Haroutunian drafted and revised the manuscript.

## Conflict of Interest Statement

The authors declare that the research was conducted in the absence of any commercial or financial relationships that could be construed as a potential conflict of interest.
